# Children’s psychological and behavioral responses following pediatric intensive care unit hospitalization: the caring intensively study

**DOI:** 10.1186/1471-2431-14-276

**Published:** 2014-10-26

**Authors:** Janet E Rennick, Geoffrey Dougherty, Christine Chambers, Robyn Stremler, Janet E Childerhose, Dale M Stack, Denise Harrison, Marsha Campbell-Yeo, Karen Dryden-Palmer, Xun Zhang, Jamie Hutchison

**Affiliations:** The Montreal Children’s Hospital, McGill University Health Centre, 2300 Tupper Street, Montreal, Quebec Canada; McGill University, 3506 University Street, Montreal, Quebec Canada; Dalhousie University, 5869 University Avenue, Halifax, Nova Scotia Canada; The IWK Health Centre, 5850-5980 University Avenue, Halifax, Nova Scotia Canada; Lawrence S. Bloomberg Faculty of Nursing, University of Toronto, 155 College Street, Toronto, Ontario Canada; The Hospital for Sick Children, 555 University Avenue, Toronto, Ontario Canada; Center for Bioethics and Social Sciences in Medicine, University of Michigan, 2800 Plymouth Road, Building 16, Room 400S-24, Ann Arbor, Michigan USA; Department of Psychology and Centre for Research in Human Development, Concordia University, 7141 Sherbrooke Street West (L-PY-170), Montreal, Quebec Canada; Children’s Hospital of Eastern Ontario, 401 Smyth Road, Ottawa, Ontario Canada; University of Ottawa, 75 Laurier Ave East, Ottawa, Ontario Canada; Research Institute of the McGill University Health Centre, 2155 Guy Street, Montreal, Quebec Canada; The University of Toronto, 27 King’s College Circle, Toronto, Ontario Canada

**Keywords:** Pediatric intensive care, Child outcomes, Longitudinal follow-up, Psychological and behavioral characteristics, Study protocol

## Abstract

**Background:**

Pediatric intensive care unit (PICU) hospitalization places children at increased risk of persistent psychological and behavioral difficulties following discharge. Despite tremendous advances in medical technology and treatment regimes, approximately 25% of children demonstrate negative psychological and behavioral outcomes within the first year post-discharge. It is imperative that a broader array of risk factors and outcome indicators be explored in examining long-term psychological morbidity to identify areas for future health promotion and clinical intervention. This study aims to examine psychological and behavioral responses in children aged 3 to 12 years over a three year period following PICU hospitalization, and compare them to children who have undergone ear, nose and/or throat (ENT) day surgery.

**Methods/Design:**

This mixed-methods prospective cohort study will enrol 220 children aged 3 to 12 years during PICU hospitalization (study group, n = 110) and ENT day surgery hospitalization (comparison group, n = 110). Participants will be recruited from 3 Canadian pediatric hospitals, and followed for 3 years with data collection points at 6 weeks, 6 months, 1 year, 2 years and 3 years post-discharge. Psychological and behavioral characteristics of the child, and parent anxiety and parenting stress, will be assessed prior to hospital discharge, and again at each of the 5 subsequent time points, using standardized measures. Psychological and behavioral response scores for both groups will be compared at each follow-up time point. Multivariate regression analysis will be used to adjust for demographic and clinical variables at baseline. To explore baseline factors predictive of poor psychological and behavioral scores at 3 years among PICU patients, correlation analysis and multivariate linear regression will be used. A subgroup of 40 parents of study group children will be interviewed at years 1 and 3 post-discharge to explore their perceptions of the impact of PICU hospitalization on their children and enhance our understanding of findings generated from standardized measures in the larger cohort study. An interpretive descriptive approach will guide qualitative data collection and analysis.

**Discussion:**

This study aims to generate new information regarding the magnitude and duration of psychological and behavioral disturbances among children admitted to PICUs, potentially leading to remedial or preventive interventions.

## Introduction & rationale

Pediatric intensive care unit (PICU) hospitalization places children at increased risk of persistent psychological and behavioral problems following discharge. More than 210,000 children are admitted to PICUs in North America every year [1, 2]. Despite tremendous advances in the development of sophisticated medical technologies and treatment regimes, approximately 25% of children demonstrate negative psychological and behavioral responses within the first year post-discharge [3–5]. While PICU outcome research has historically focused on physical recovery and predictors of child mortality, research over the past 3 decades has increasingly focused on psychological and behavioral responses [4, 6–13]. Parents describe decreases in children’s self-esteem and emotional well-being, increased anxiety, and negative behavioral changes (e.g., sleep disturbances, social isolation) post-PICU discharge. School-aged children report delusional memories and hallucinations, increased medical fears, anxiety, changes in friendships and in their sense of self [5]. Psychiatric syndromes, including post-traumatic stress disorder and major depression, have been diagnosed [5, 14]. These studies have generally been conducted in the first year post-PICU discharge with the majority assessing symptoms within the first 6 months [5, 14, 15]. However, health-related quality of life (HRQoL) studies suggest that deterioration in children’s emotional well-being may be longer-lasting [16–20]. In fact, there is a near-complete absence of data tracking the 12- to 36-month period following PICU hospitalization. We do not know whether early sequelae persist, diminish, or worsen over time. Furthermore, children under the age of 6 years who constitute the bulk of the PICU population have rarely been included in research to date, suggesting the incidence of negative psychological and behavioral responses may be greatly underestimated [14, 21, 22].

While psychological well-being in children is comprised of a number of interrelated factors (an absence of psychological symptoms, participation in age-appropriate tasks and activities within the family and broader community, and feelings of positive self-esteem [23]), this is not reflected in the PICU literature. Rather, studies have focused primarily on psychological outcomes specific to a particular psychiatric disorder (e.g., post-traumatic stress disorder), despite research suggesting this approach is limited [14, 24]. Indeed, there have been no systematic attempts to understand the broad alterations in children’s psychological well-being as they recover and return to normal activities post-PICU. Within the field of childhood critical illness, our understanding of children’s psychological and behavioral responses remains in its infancy, as does our repertoire of appropriate interventions. To impact recovery in this population, it is essential to enhance our understanding of the magnitude and duration of potentially harmful emotional and behavioral changes in these children, and to identify clinical risk and protective factors such as child and parent characteristics associated with sustained psychological change.

Our mixed-methods prospective cohort study will enrol children aged 3 to 12 years and their parents prior to PICU discharge from three Canadian pediatric hospitals, and follow them over a 3-year period. The study is based on an integrative model of pediatric medical traumatic stress [25], and was designed to address important gaps in the field. Specifically, we will examine a broader array of age-appropriate, psychological and behavioral response indicators than have been used previously, explore parents’ and children’s perceptions of their well-being, and identify risk factors that may impact post-PICU recovery. Study results will provide new knowledge about the magnitude and duration of psychological and behavioral responses among children admitted to PICUs, potentially leading to remedial or preventive interventions.

## Background

### Children’s psychosocial outcomes following PICU hospitalization

Critical illness exposes children to extreme stressors. These include highly invasive procedures, separation from families, other critically ill and dying children, altered levels of consciousness, elevations in light and noise levels, and multiple strangers providing sophisticated caretaking procedures. These children demonstrate significant sleep loss and frequent awakenings [26–28]. Descriptive studies examining school-aged children’s retrospective perceptions of the PICU have found that they perceive it as highly anxiety-provoking and demonstrate distortion in their recall of events [29–33]. Up to 63% of children (n = 102) have been found to recall some aspect of their PICU stay [3], including medical procedures [34], endotracheal intubation [3, 35], and pain [3]. Parents have described behavioral changes and ongoing fears in their children years after PICU discharge [36], as well as changes in children’s memory, attention span, cognitive functioning, self-esteem and self-confidence [6].

In a controlled prospective cohort study, we found younger children (n = 60 PICU; n = 60 Ward) who were more severely ill and exposed to higher numbers of invasive procedures demonstrated elevated medical fears (17%) and symptoms of post-traumatic stress (25%) 6 months post-discharge [11]. PICU children were exposed to a fourfold increase in invasive procedures compared to children on medical and surgical wards. The number of invasive procedures was subsequently identified as the most important predictor of negative psychological outcomes post-PICU discharge [12]. Other studies have identified high baseline levels of children’s externalizing and internalizing behaviors as significant predictors of negative behaviors 6 months post-discharge [37, 38]. Heightened PICU maternal state anxiety was also a significant predictor of heightened child anxiety and externalizing behaviors 3 months post-discharge [38].

In our earlier studies, we interviewed children aged 6 to 12 years 3 months post-PICU discharge, their parents, and health care professionals (n = 52) to inform the development of two new child self-report measures of post-PICU psychological distress: the Children’s Critical Illness Impact Scale, written version (CCIIS©), and the Young Children’s Critical Illness Impact Scale, pictorial version (Y-CCIIS©) [22, 24, 39]. Parents described children’s behavioral changes and heightened anxiety, while children expressed anxiety, fears, changes in relationships with family and friends, and changes in their sense of self. How these psychological and behavioral responses change over time or are influenced by other factors remains unknown. Given that factors comprising psychological well-being are multiple and interrelated, alterations in the child’s sense of self and interpersonal relationships have the potential to impact recovery during the early post-discharge period, and during critical periods of growth and development. The notion of sustained negative effect is supported by health-related quality of life (HRQoL) studies that identify deterioration in the emotional well-being of 20% to 30% of children up to 1 year post-PICU discharge, despite demonstrating little or no change in overall quality of life [9, 16, 18, 19]. In the only study to follow children beyond 1 year post PICU discharge, 16.4% of participants reported unfavorable HRQoL (n = 727, 0 to 29 years of age, hospitalized as children) an average of 3.5 years post-PICU admission [20]. While HRQoL measures provide limited information on psychological health, these results suggest that the consequences of PICU admission may be long-lasting.

A systematic review of children’s psychological outcomes following PICU hospitalization grouped studies (n = 28) into four categories: PICU perceptions and recall, psychological outcomes, post-traumatic stress symptoms (including post-traumatic stress disorder, or PTSD), and general health status and quality of life [5]. Studies conducted during the past decade have focused primarily on outcomes specific to a particular psychiatric disorder, most commonly PTSD. Critical illness is, by definition, life threatening, and since psychiatric disorders can be triggered by exposure to extreme stressors in a vulnerable population, critical illness can present serious threats to children’s long-term health and well-being [14, 40]. While PICU hospitalization increases the risk for post-traumatic stress symptoms such as irritability, avoidance of situational reminders of the hospital experience, anxiety and depression, psychiatric disorders such as PTSD are diagnosed less frequently [4, 7, 10–12, 14, 15, 32, 41]. The use of diagnostic frameworks, which call for a dichotomous report of the presence or absence of the condition being examined, may have resulted in an underestimation of the extent of children’s psychological and behavioral problems post-PICU. This was supported in our measurement development research [22, 24, 39].

Advances in technologies and surgical techniques continue to alter the composition of the PICU population such that the majority of children are now less than 6 years of age. Yet younger children remain largely excluded from psychological outcome studies, raising important concerns about the psychological impact of critical illness on this segment of the population [5, 14]. Preschoolers may be excluded as they are more difficult to assess, and because of a lack of instrument validation with this age group. Thus, it is important to consider developmental differences when assessing psychological outcomes across a broad range of age groups to collect accurate information. This study will assess emotional and behavioral responses following a PICU stay or ENT day surgery in children as young as 3 years, using developmentally appropriate measures.

### Conceptual model of pediatric medical traumatic stress

**Figure 1:**
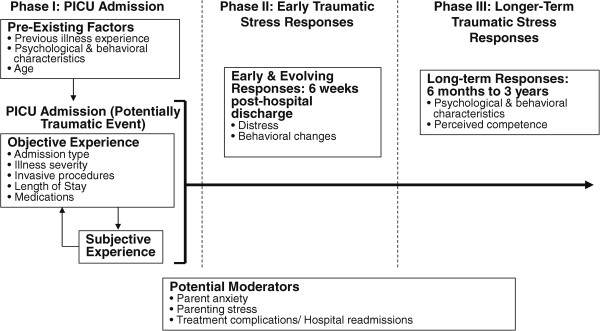
**An integrated model of pediatric medical traumatic stress (Adapted with permission from Kazak et al 2006).**

An integrative model of pediatric medical traumatic stress (PMTS) guides the study (Figure 1) [25]. PMTS is defined as a set of psychological and physiological responses to pain, injury, serious illness, medical procedures, and invasive or frightening treatment experiences [42]. We propose that each phase of the model represents part of the PICU admission and recovery process, and present factors identified in the literature that may influence outcome, and that are included in our study. Since children’s psychological responses are likely to be influenced by factors that evolve over the course of follow-up, we will gather data on parenting stress, and the child’s own evolving psychological and behavioral responses, as well as illness or treatment complications and further hospitalizations that may precipitate distress [25, 43]. Normal developmental transitions from preschool to middle childhood and middle childhood to adolescence, as well as entry to formal schooling and the transition from elementary to high school will be documented. All of these factors contribute to the developing child’s sense of self, as well as mechanisms of cognitive and emotion regulation that ultimately influence psychological adjustment [25].

### Summary

Negative psychological outcomes have been identified in approximately 25% of children in the first year post-PICU discharge [3–5, 15]. The magnitude of the problem may be largely underestimated due to a paucity of research with children under 6 years of age, and the widespread use of psychiatric diagnostic frameworks to capture what appears to be a broader array of psychological and behavioral symptoms. In view of the near complete absence of data during the 12- to 36-month period post-discharge, we will conduct the first prospective cohort study to longitudinally examine psychological and behavioral responses in 3- to 12-year-old children over the 3-year period following PICU hospitalization. This important and innovative study will consider the complexity of the developing child while identifying factors that may influence long-term responses to medical traumatic stress. Ultimately, we propose to translate this knowledge into future remedial or preventive interventions aimed at fostering healthy child development.

## Objectives and specific hypotheses

### Primary objective

1. To examine children’s psychological and behavioral responses as measured by the Behavioral Assessment System for Children (BASC-2) at 6 months, 1 year, 2 years and 3 years post-PICU hospitalization, and compare them to those of children who have undergone minor ENT day surgery.

### Secondary objectives

2. To examine children’s behavioral responses and their psychosocial impact at 6 months, 1, 2, and 3 years post-PICU hospitalization using the Strengths and Difficulties Questionnaire (SDQ), and compare them to those of children who have undergone ENT day surgery.
3. To examine children’s perceptions of self-competence at 6 months, 1, 2, and 3 years post-PICU hospitalization using the Harter Scale of Perceived Competence (Harter), and compare them to those of children who have undergone ENT day surgery.
4. To identify predictors of children’s psychological and behavioral responses at 6 months, 1, 2, and 3 years post-PICU hospitalization.
5. To explore a subgroup of parents’ perceptions of the impact of PICU hospitalization on children at 1 and 3 years post-discharge.

### Hypotheses

The following hypotheses are proposed in relation to objectives 1 through 4. Objective 5 will be achieved using qualitative interpretive methods.

a) Children will continue to demonstrate more negative psychological and behavioral responses 3 years post-PICU discharge than post-ENT day surgery; b) Group differences will peak at 1 year, and become smaller over time (years 2 and 3); c) Psychological and behavioral difficulties will peak 6 months post-PICU discharge, remain stable to 1 year, then slowly decline over years 2 and 3, remaining significantly higher than those experienced post-ENT day surgery.
Children will have more overall problem behaviors at 6 months, 1, 2, and 3 years post-PICU discharge than post-ENT day surgery.
Children will have lower levels of perceived competence at 6 months, 1, 2, and 3 years post-PICU discharge than following ENT day surgery.
Children’s psychological and behavioral responses 6 months, 1, 2, and 3 years post-PICU will be related to child baseline factors (age, number of previous hospitalizations, psychological and behavioral characteristics); PICU-based factors (length of stay, illness severity, invasive procedures, parent anxiety); child distress 6 weeks post-discharge; and parenting stress, life stresses, hospital re-admissions, cumulative numbers of invasive procedures, and professional psychological counseling over the course of follow-up.

## Methods/Design

### Design

**Figure 2:**
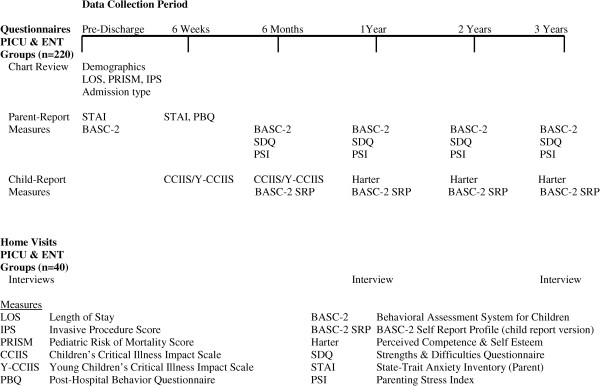
**Mixed methods study design.**

A mixed-methods prospective cohort design will be used to examine children’s psychological and behavioral responses over a 3-year period post-PICU hospitalization (study group n = 110), and post-ENT day surgery (comparison group n = 110). A subgroup of parents of children in the study group (n = 20) and the comparison group (n = 20) will also be interviewed at 1 and 3 years post-discharge using an interpretive descriptive approach [44, 45]. Parents will be invited to include their child for a portion of each interview to allow a fuller understanding of the impact of hospitalization. The complementarity of the quantitative and qualitative data will provide a comprehensive understanding of children’s psychological and behavioral responses to PICU hospitalization [46]. We will use standardized questionnaires with all participants (n = 220). With open-ended interviews we will explore a subgroup of participants’ (n = 40) perceptions of the impact of PICU and day-surgery hospitalization on the child at 1 year (close to the study mid-point), and 3 years (the final data collection point) post-discharge. This will allow us to probe participant responses to questionnaire items [45, 47, 48]. In addition, interviewing parents in both groups will allow us to capture a broad scope of hospital experiences. Multiple types of data will enhance the potential for developing new insights in the field and exploring alternative interpretations of the data, ultimately enhancing data validation [47, 49]. Data will be collected throughout the follow-up period (Figure 2). To ensure sufficient time for enrolment and follow-up, this study will take place over a 5-year period across 3 Canadian pediatric hospitals.

An ENT day-surgery comparison group was chosen for several reasons. They are an otherwise healthy group of children who have nominal contact with the medical system. They will undergo a hospitalization experience resulting in a minimal length of stay, low invasive procedure scores, and low severity of illness scores—variables to which our PICU group will have higher exposure. In addition, they will act as controls for expected maturational changes in children over the course of the 3-year follow-up.

### Setting

The study will take place in 3 university-affiliated pediatric hospitals located across 3 Canadian provinces: The Montreal Children’s Hospital (MCH) in Montreal, Quebec, The Hospital for Sick Children (HSC) in Toronto, Ontario, and the IWK Health Centre (IWK) in Halifax, Nova Scotia. All hospitals have PICUs that admit children from infancy to 17 years with a wide range of medical and surgical diagnoses. Comparison groups will be drawn from hospital ENT day surgery units at the MCH and the IWK.

Study recruitment will take place in the PICU (study group). The first data collection point post-PICU will be on the hospital ward prior to discharge. Children in the comparison group will be recruited through the hospital day surgery unit. The first data collection point will be at the hospital following surgery. Data will subsequently be collected using standardized measures that parents complete at home. Parents asked to participate in follow-up interviews will be purposively selected, as we wish to recruit a heterogeneous sample that reflects the general PICU (and ENT) population. Interviews will take place following questionnaire completion at years 1 and 3 in the families’ homes, or at the study site from which they were recruited, based on parent preference.

### Population

The target population is children aged 3 to 12 years who have suffered a critical illness requiring PICU admission for a minimum of 24 hours, and are expected to survive. Children must speak English or French to participate, one parent must speak, read and write English or French, and the child must be identified as ready for discharge. While the PICU population is heterogeneous, medical diagnoses have not been related to psychological sequelae. Rather, illness severity and exposure to invasive procedures have been identified as significant predictors of post-traumatic stress symptoms 6 months post-discharge [11]. To capture the full range of admissions, we will only exclude children who have had a previous PICU admission, and those who suffer severe brain injury and neurological impairment (because they cannot be evaluated using standardized measures). On the day we begin recruiting at each site (i.e., at study inception only), we will exclude children who have been in the PICU longer than 60 days (1.1% of MCH and 0.96% of HSC admissions in 2011), as this will preclude our ability to gather baseline BASC-2 scores. Afterwards, all children, regardless of admitting diagnosis and length of stay, and their parents (or primary caretaker) will be potential candidates.

The comparison group will include children aged 3 to 12 years who have undergone ENT day surgery. Children must speak English or French, and one parent must speak, read and write English or French to participate. Any imbalance that may occur between groups will be adjusted for in the statistical analyses. Children who have experienced a previous PICU admission, or who are neurologically impaired will be excluded from the comparison group (as per the study group). We will not exclude children in the comparison group who require PICU hospitalization during the 3-year follow-up period, because this would result in an overly healthy group at baseline. Rather, we will censor these children at the time of PICU admission in order to retain information about them for as long as possible in the study [50].

### Procedures

All PICU admissions and those booked for ENT day surgery will be screened for eligibility. A Research Assistant (RA) will approach parents of eligible children to explain the study, answer questions, and obtain parent consent and child assent from those who agree to participate. A subgroup of 20 PICU and 20 ENT parents will be invited to participate in face-to-face interviews at year 1 and 3 follow-up points. All parents will be asked at enrolment if they would agree to be contacted regarding participation in these interviews, and 40 parents will be subsequently be selected (see Sample size estimation). Following enrolment, demographic and hospitalization data will be collected from the child’s medical chart and verified with parents. Hospitalization information will include medical diagnosis, number of previous hospitalizations, length of stay, pediatric risk of mortality (PRISM-3 [51]) score as an indicator of illness severity, and the child’s invasive procedure score (IPS [11]). Parents will complete the State-Trait Anxiety Inventory (STAI [52, 53]) as an indicator of parent anxiety, and the BASC-2 [54] as an indicator of their child’s usual (i.e., pre-admission) emotional and behavioral characteristics. PICU admissions are frequently unplanned, making it impossible to gather data prior to admission. The BASC-2 items ask about child characteristics over the previous 6 months; therefore, parents will complete it immediately following their child’s PICU or day surgery stay, and will be asked to think about their child’s usual behavior during the 6-month period prior to hospitalization. For children who remain in the PICU 60 days post-admission and who are not ready for discharge, parents will complete the BASC-2 at that time to minimize recall bias. In this way, we expect all baseline scores to reflect the child’s usual pre-hospital characteristics.

The second period of data collection will take place 6 weeks post-PICU or day surgery hospitalization (Figure 2). Parents will complete two questionnaires requiring a total of 20 to 30 minutes: the STAI, and the Post-Hospital Behavior Questionnaire (PHBQ [55, 56]), which evaluates changes in the child’s behavior since hospitalization. Children aged 6 years and older will complete the Children’s Critical Illness Impact Scale, written (CCIIS [24, 57]) or pictorial (Y-CCIIS [39]) version depending on reading ability, which takes 10 to 20 minutes to complete. Parents will receive follow-up telephone calls from the site-based RA to ensure questionnaires were received, answer any questions, and encourage parents to return them by mail. Those who do not return questionnaires within 3 weeks will be offered the possibility of a home courier pick-up.

Data collection will continue at 6 months, 1, 2, and 3 years post-discharge (Figure 2). Reminder telephone calls will ensure complete follow-up, and parents will be asked whether their child has required further hospital admissions. The IPS (based on chart review) will be calculated for each readmission, and a cumulative (follow-up) IPS score will be generated. Parents will be asked to spend approximately 45 minutes at each follow-up point completing 3 questionnaires: the BASC-2; the Parenting Stress Index (PSI [58]) to assess stress in the parent–child system as well as life stresses beyond parental control; and the SDQ [59, 60], which screens child behavior and its impact on the child and others. Children will complete the CCIIS again at the 6-month visit to measure distress post-discharge. Children aged 8 years and older will complete the BASC-2 Self-Report Profile (SRP), an indicator of their usual emotional and behavioral characteristics.

Questionnaires will be mailed back to participants’ respective recruitment sites, allowing site-based RAs to keep track of mailings and follow-up requirements. The BASC-2 (primary outcome variable) has clinical cut-off scores, and while scores cannot be interpreted in isolation (the child would require further assessment by a clinical psychologist or psychiatrist), parents will be informed if their child’s behavioral symptoms index composite score falls within a clinically significant range; specifically, if the child receives a t-score >70 on either the Parent Report Scale or the Child Self Report Scale (i.e. scores ≥95% percentile on the overall behavioral symptoms index of the parent version, and the emotional symptoms index of the child version). Parents will receive a telephone follow-up call, a letter explaining their child’s score, and information regarding community resources they can contact for support. We will track care received through professional mental health services, and explore potential effects in our analyses should changes in children’s patterns of recovery be observed.

Face-to-face, 1-hour interviews will take place at the end of years 1 and 3. At the initial interview, the RA will ask open-ended, semi-structured questions designed to elicit a narrative from parents about their experience of events and their perceptions of their child’s return to daily life. Interviewers will also probe parents’ responses to questionnaire items. At the second interview, semi-structured questions will be tailored to participants’ previous interview and questionnaire responses. At each interview, parents will be encouraged to invite their child to be present for the first 20 to 30 minutes to comment on their experiences of returning to daily life after hospitalization. The purpose of including the child is to obtain a fuller understanding of the impact of hospitalization. The interview focus will then shift to include the parents only so that they have an opportunity to share their own narratives without the child present. Parents’ wishes to include or exclude their child will be respected.

### Measures

All measures have been tested for their psychometric properties and translated from English into French. The primary outcome measure of children’s psychological and behavioral responses following hospitalization is the BASC-2. The BASC-2 assesses positive (adaptive) and negative (clinical) dimensions of emotional (e.g., anxiety, depression, somatisation), behavioral (e.g., hyperactivity, withdrawal, aggression), and adaptive functioning (e.g., social skills, leadership, adaptability) in children aged 2 ½ to 18 years [54]. It is a multidimensional measure with excellent psychometric properties, and is well-suited to the heterogeneous population of children admitted to the PICU.

Secondary outcome measures include the SDQ, which characterizes children’s behavioral responses and contains an impact supplement that provides complementary information [59, 60]. Parents are asked whether they feel their child has an emotional, concentration or behavioral problem and, if so, to describe the chronicity, distress, social impairment and burden to others of that problem. The Harter Scales of Perceived Competence (referred to as Harter, hereafter) provide self-report information on perceived competence from the child’s perspective, reflecting an essential component of psychological well-being [61, 62].

Predictors of children’s psychological and behavioral responses following hospitalization include child, parent and hospitalization characteristics. We will measure child characteristics at baseline including age, number of previous hospitalizations, and previous emotional and behavioral characteristics (using the BASC-2 as an indicator of pre-admission behavior), as well as distress 6 weeks post-discharge (CCIIS [24, 57] and PHBQ [55, 56]). Parent anxiety will be measured in hospital and at 6 weeks post-discharge (STAI [52, 53]). Data regarding illness severity (PRISM-3 [51]), number of invasive procedures the child is exposed to (IPS [11]), and length of stay will be gathered during hospitalization. These variables may be used to identify high risk children.

Potential mediators of children’s long-term responses include parenting stress and other life stresses (PSI [58]), number of hospital readmissions, cumulative number of invasive procedures (IPS [11]) related to hospital readmissions, and number of professional counselling sessions during follow-up.

### Data analysis

In this mixed-methods study, quantitative data will be analyzed using SAS version 9.3 (SAS Institute, Cary, NC). To describe the two groups of participants, demographic and clinical characteristics at baseline will be summarized using means or medians for continuous data and proportions for categorical data. Psychological and behavioral response scores for PICU and ENT groups will be calculated and compared at each follow-up time point, both statistically and graphically.

To address our primary objective, we will compare group response scores on the BASC-2 at 3 years post-hospitalization, as well as at 6 months, 1 year and 2 years post-hospitalization using independent two-sample T-tests. We will then compare psychological and behavioral responses within patients and between groups at 6 months, 1, 2, and 3 years post-discharge using a mixed effect linear regression model to take the random effect of time into account and to adjust for baseline characteristics [63].

To address our secondary objectives, we will: (a) compare children’s problem behaviors on the SDQ within patients and between groups at 6 months, 1, 2 and 3 years post-hospitalization using a mixed effect linear regression model to take the random effect of time into account and to adjust for child characteristics at baseline; (b) compare children’s perceived competence on the Harter within patients and between groups at 6 months, 1, 2 and 3 years post-hospitalization using a mixed effect linear regression model to take the random effect of time into account and to adjust for child characteristics at baseline; (c) explore baseline child and PICU-based factors and potential mediators predictive of poor psychological and behavioral response scores 6 months, 1, 2 and 3 years post-PICU hospitalization using correlation analysis and multivariate linear regression. Statistical analyses will be conducted following the verification of assumptions underlying those analyses. In our previous work, PICU children were categorized as high or low risk in a secondary data analysis based on a positive skew in the distribution of invasive procedure score data [12]. We do not wish to assume a similar distribution based on changes in the PICU population over the past decade, and study limitations identified in our previous secondary data analysis; therefore, we will examine the data and determine whether non-linear terms or transformations are needed in the regression models.

To address our final secondary objective, qualitative (interview) data will be analyzed concurrently with quantitative and qualitative data collection. This will foster data complementarity by allowing us to probe questionnaire responses [46, 64], generate data that cannot be captured by questionnaire measures alone [46], and ultimately produce deeper insights into children’s psychological and behavioral responses to PICU hospitalization [49]. Audiotapes of interviews will be transcribed verbatim, read in their entirety, and coded line-by-line. Open-coding will be used with the initial semi-structured interviews to identity themes in participants’ narratives [65]. Prominent themes will be flagged for incorporation into the second semi-structured interview, along with items of concern identified on the completed questionnaires. Semi-structured scripts for the second interview will be tailored to themes identified in the first interview and the questionnaire items we wish to probe further. We will use open and axial coding with second interviews to connect the emergent categories [65]. For all interviews, data will be analyzed using the constant comparison method [66]. Comments by children present during the interviews will be highlighted. NVivo software will be used to support data management and sorting [67].

### Sample size estimation

The sample size estimate is based on psychosocial and behavioral response scores on the BASC-2 at 3 years post-PICU discharge. A sample size of 126 participants (63/group) will achieve 80% power at a 0.05 significance level, allowing us to detect a medium effect size of 0.5 between groups using an independent two-sample T-test. Baseline child characteristics may differ in PICU and ENT groups; therefore, we will adjust for potential confounding using multivariate linear regression and, in anticipation of this, have added an additional 20% to our sample size. Finally, we estimated a 30% attrition rate at 3 years (attrition in our 2002 cohort study was 13% at 6 months [11]). Therefore, we aim to enroll no fewer than 110 children per group (n = 220).

We will conduct interviews with a subset of 40 parents (20/group) of children enrolled in the larger cohort. Purposive sampling will be used to select parents whose children reflect the heterogeneous group normally admitted to the PICU, as well as children admitted for typical ENT day surgeries. We anticipate this sample size will allow for a fuller understanding of children’s experiences [46, 49], and allow us to reach data saturation [47].

#### Ethical considerations

The study and all consent and assent forms were reviewed by the Research Ethics Boards of participating institutions. Participants are asked to provide written consent (parent), and verbal or written assent (child) according to provincial law. They can withdraw from the study at any time during the 3-year follow-up period without affecting patient care. Parents of children whose overall score on the BASC-2 falls within a clinically significant range (≥95% percentile on the overall behavioral or emotional symptoms index) at any of the follow-up time points will be informed and directed to local community health resources.

#### Knowledge dissemination

A report of the study findings will be shared with all participants, and they will be invited to a presentation of the findings. We will submit a copy of the report and invitations to the presentation of study findings to the parent/child councils and family support groups in participating hospitals and community partners, including rehabilitation and transition facilities. To promote broader community engagement, findings will be shared with clinicians and researchers at all sites, and telehealth presentations will facilitate dissemination to a larger professional audience. Study findings will be published in peer-reviewed journals and disseminated via academic meetings.

## Discussion

Our study will be the first mixed-methods, multi-site study to follow children aged 3 to 12 years longitudinally for 3 years post-PICU hospitalization. The study is innovative in three ways. First, the mixed-methods design is expected to produce substantial, nuanced data on the trajectory of children’s psychological and behavioral responses to PICU hospitalization and their long-term recovery. This will help us understand what long-term problems we might anticipate with this population, and identify potential risk and protective factors, including those amenable to clinical interventions. Second, this will be the first study to focus on preschoolers in addition to school-aged children. Third, the study will move beyond the predominant focus on PTSD as the primary psychological outcome in post-PICU hospitalization research, by examining the interaction of multiple risk factors on a broader array of psychological and behavioral outcomes over time. Study findings are expected to lead to several promising avenues of research. They will offer opportunities to design and test clinical interventions for young children. We expect to identify clinical characteristics and child- and parent-related factors that will facilitate our ability to recognize children at risk, and to develop interventions targeted at factors such as parent anxiety and child distress in the early post-discharge period. Results from this study will produce new knowledge in a previously unexplored area, with potential for high impact in a growing area of novel childhood experience.

## Abbreviations

BASC-2: Behavioral Assessment System for Children, version 2
CCIIS: Children’s Critical Illness Impact Scale
ENT: Ear, nose and/or throat
HRQoL: Health-related quality of life
HSC: Hospital for Sick Children
IPS: Invasive procedure score
IWK: IWK Health Center
MCH: Montreal Children’s Hospital
PHBQ: Post-Hospital Behavioral Questionnaire
PICU: Pediatric intensive care unit
PMTS: Pediatric medical traumatic stress
PRISM-3: Pediatric risk of mortality score, version 3
PSI: Parenting Stress Index
PTSD: Post-traumatic stress disorder
SDQ: Strengths and Difficulties Questionnaire
SRP: Self-Report Profile
STAI: State-Trait Anxiety Inventory
Y-CCIIS: Young Children’s Critical Illness Impact Scale.
